# lncRNA-TINCR Functions as a Competitive Endogenous RNA to Regulate the Migration of Mesenchymal Stem Cells by Sponging miR-761

**DOI:** 10.1155/2020/9578730

**Published:** 2020-02-26

**Authors:** Jiaqian Zheng, Yanni Huang, Ying Li, Jieqing Lai, Chen Chen, Chunzhi Yi, Fengxiang Pang, Yun Lan, Liangliang Xu, Bin Fang

**Affiliations:** ^1^Key Laboratory of Orthopaedics & Traumatology, The First Affiliated Hospital of Guangzhou University of Chinese Medicine, Guangzhou University of Chinese Medicine, Guangzhou, China; ^2^Laboratory of Orthopaedics & Traumatology, Lingnan Medical Research Center, Guangzhou University of Chinese Medicine, Guangzhou, China; ^3^The Research Center of Basic Integrative Medicine, Guangzhou University of Chinese Medicine, Guangzhou, China

## Abstract

Mounting evidences have indicated that terminal differentiation-induced lncRNA (TINCR) contributes to various cellular processes, such as proliferation, apoptosis, autophagy, migration, invasion, and metastasis. However, the function of TINCR in regulating migration of MSCs is largely unknown. In this study, the effects of TINCR on the migration of rat MSCs from the bone marrow were studied by Transwell assays and wound healing assays. Our results suggested that TINCR positively regulated migration of rMSCs. miR-761 mimics suppressed rMSC migration, whereas miR-761 inhibitor promoted migration. Target prediction analysis tools and dual-luciferase reporter gene assay identified Wnt2 as a direct target of miR-761. miR-761 could inhibit the expression of Wnt2. Further, the investigation about the function of TINCR in miR-761-induced migration of rMSCs was completed. These results demonstrated that TINCR took part in the regulation of miR-761-induced migration in rMSCs through the regulation of Wnt2 and its Wnt2 signaling pathway. Taken together, our results demonstrate that lncRNA-TINCR functions as a competitive endogenous RNA (ceRNA) to regulate the migration of rMSCs by sponging miR-761 which modulates the role of Wnt2. These findings provide evidence that lncRNA-TINCR has a chance to serve as a potential target for enhancing MSC homing through the miR-761/Wnt2 signaling pathway.

## 1. Introduction

Long noncoding RNAs (lncRNAs) are functional RNAs that lack protein-coding ability, which is more than 200 nucleotides in length. To date, numerous studies have revealed its important roles in cellular processes, such as X chromosome inactivation, splicing, imprinting, epigenetic control, and gene transcription regulation [1–3]. Moreover, some have shown that lncRNAs could function as a competing endogenous RNA (ceRNA) to sponge miRNAs, thereby preventing microRNAs from binding to their target genes [4–7].

A series of lncRNAs have been identified to be crucial regulators in the development and progression of cancers [8, 9]. TINCR, a terminal differentiation-induced lncRNA with 3.7 kb transcript, is discovered from human well-differentiated somatic tissues [10]. There is growing evidence that TINCR is involved in various cellular processes, such as proliferation, apoptosis, autophagy, migration, invasion, and metastasis [11–14]. For instance, Xu et al. found that the overexpression of TINCR regulates cell proliferation and apoptosis by affecting KLF2 mRNA stability in gastric cancer (GC) [15]. In addition, TINCR has been reported to function like a “sponge.” Chen et al. found that TINCR regulates cell apoptosis and proliferation of GC cell by sponging miR-375. Yu et al. discovered that TINCR could sponge miR-7-5p to promote colorectal cancer (CRC) progression. Liu et al. identified that TINCR functions as ceRNA contributing to non-small-cell lung cancer (NSCLC) proliferation. A similar study conducted by Liu et al. showed that aberrantly upregulated TINCR stimulates tumorigenesis in breast cancer, via competing with miR-7. Nevertheless, the role of TINCR in MSCs is largely unknown. Whether and how microRNAs are involved in it is also waiting for demonstration [5, 16–18].

Mesenchymal stem cells (MSCs) are nonhematopoietic cells that exist in many different tissues [19, 20]. To date, researchers focus on the mechanism of MSC migration with the prosperity of stem cell therapy and regenerative medicine outcomes [21]. In the current study, we show that TINCR is a positive regulator of rMSC migration. miR-761 mimics suppressed rMSC migration, while miR-761 inhibitor promoted migration. Furthermore, mechanistic analysis revealed that lncRNA-TINCR could function as a competitive endogenous RNA (ceRNA) to regulate the migration of rMSCs by sponging miR-761 which modulates the role of Wnt2. These findings provide evidence that lncRNA-TINCR may serve as a potential prognostic marker and therapeutic target for those MSC-relevant cancers progressing through the miR-761/Wnt2 signaling pathway.

## 2. Materials and Methods

### 2.1. rMSC Isolation and Culture

This experiment was approved by the Animal Care and Use Committee of Guangzhou University of Chinese Medicine. The method of rMSC isolation and cultivation has been described previously [22]. Shortly, first, Sprague–Dawley rats (male, 4 weeks old, 60–80 g) were chosen to detach their bilateral femur. Second, using *α*-MEM (HyClone) with 10% FBS (Gibco) and 1% penicillin-streptomycin solution (HyClone), the bone marrow of the bilateral femur was flushed out. Then, the obtained single-cell suspension was cultured at 37°C with 5% CO_2_. rMSCs from passage 3 or 5 were used for analysis.

### 2.2. Real-Time PCR

Following the manufacturer's manual, the total RNA was extracted from bone marrow-derived rMSCs using Takara MiniBEST Universal RNA Extraction Kit (Takara), and the quantity and purity were measured by NanoDrop 2000 subsequently. The following operation all complied with the corresponding manufacturer's instructions. For TINCR, 1 *μ*g total RNA was reversely transcribed into cDNA by PrimeScript RT Master Mix (Takara). For Wnt2, cDNA was synthesized with miRcute Plus miRNA First-Strand cDNA Kit. The quantitative PCR was performed on ABI Prism 7900 HT. All primers used in this study were listed in Table 1.

### 2.3. Oligonucleotide Synthesis and Transfections

The oligonucleotides, like siTINCR and miR-761 mimics and inhibitor, were synthesized by GenePharma (Shanghai, China). Before the day of transfection, rMSCs were seeded into 6-well cell culture cluster with a density of 1.5 × 10^5^ cells each well. According to the manufacturer's instructions, cells were transfected with miR-761 mimics, inhibitor, or siTINCR. The cells were used after the terminal transfection for analysis 24 h later.

### 2.4. Lentiviral Vector Construction

Cloned into lentiviral vector to guarantee stable infection, the synthetic oligonucleotides used for overexpression of TINCR (lvTINCR) were also synthesized by GenePharma (Shanghai, China). According to the manufacturer's instructions, lvControl and lvTINCR were added after cell adherence. Culture medium was replaced at 24 h after transfection and used for analysis followed by cell culture for additional 4 h.

### 2.5. Transwell Assays

After transfection of miRNA-761 mimics, miR-761 inhibitor, lvTINCR, and siTINCR, respectively, cells with a density of 8 × 10^4^ cells per well were plated to the upper chamber of the Transwell chamber (Corning Costar) in a serum-free medium with 10% FBS containing the medium at the bottom layer. Incubated for 12 h at 37°C, rMSCs at the upper layer of the membrane were scraped and rMSCs at the lower layer were stained with 0.5% Crystal Violet Staining Solution and photographed under a microscope. A number of cells were quantified in the randomly selected fields.

### 2.6. Wound Healing Assays

After transfection of miRNA-761 mimics, miR-761 inhibitor, lvTINCR, and siTINCR, respectively, rMSCs were incubated in 6 cm dish and cultured until 95% confluence. A scratch wound contributes to a micropipette tip, and culture medium with 10% FBS was replaced by culture medium with 1% FBS. The cells were photographed and counted under a phase contrast microscope 12 h later.

### 2.7. Bioinformatic Analysis

The bioinformatic analysis of miRNAs was performed using TargetScan (http://www.targetscan.org) and miRDB (http://mirdb.org/). The target genes were identified using in vitro experiments.

### 2.8. Luciferase Reporter Assay

Based on the manufacturer's instructions, a luciferase reporter assay was performed using the Dual-Luciferase Reporter Assay System (Promega, Madison, WI, USA). Briefly, wild-type and mutant Wnt2 (without miR-761 binding sites) plasmids PmiR-Glo-Report™ were cotransfected with miR-761 mimics or mimics NC into 293T cells using Lipofectamine 2000 (Invitrogen). The luciferase activity was measured at 48 h after transfection using the GloMax™ 20/20 Single Tube Luminometer (Promega, Madison, WI, USA).

### 2.9. Western Blot

The proteins used for Western blot were extracted using whole cell lysates described previously [23]. The BCA Protein Assay Kit (Beyotime, Jiangsu, China) was used to quantify the protein samples. Equal proteins were loaded onto 10% SDS-PAGE. Then, all the blots were transferred onto a polyvinylidene difluoride (PVDF) membrane (Millipore). The membrane, subsequently, was blocked with 5% skim milk for 2 h at room temperature and was incubated with antibodies against *β*-catenin (Abcam, 1 : 1000) and CXCR4 (Abcam, 1 : 1000) at 4°C overnight, respectively. After the membrane was rinsed with TBST three times, horseradish peroxidase-linked secondary antibodies were used to incubate for 1.5 h at room temperature. Lastly, proteins were detected with Pierce® ECL Western Blotting Substrate (Thermo Scientific) according to the manufacturer's instruction.

### 2.10. Statistical Analysis

Data is presented for each group as means ± standard deviation (SD). Analysis was performed using SPSS 22.0 software. Differences between groups were compared by *t*-tests or one-way analysis of variance (ANOVA). *P* < 0.05 was considered to be statistically significant.

## 3. Result

### 3.1. TINCR Promoted Cell Migration

**Figure 1:**
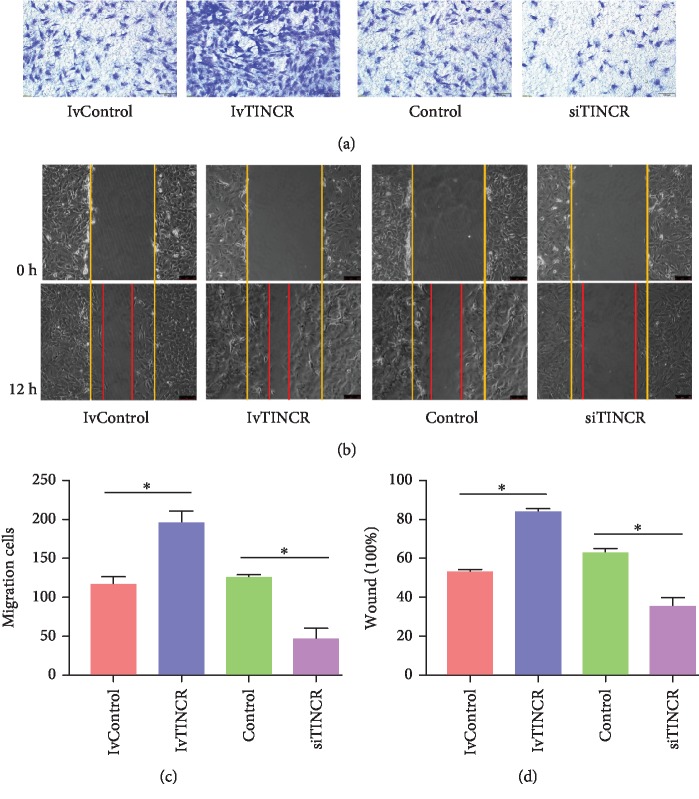
TINCR promoted cell migration. lvTINCR and siTINCR were transfected into rMSCs, respectively. (a) Transwell assays. Cells with a density of 8 × 10^4^ cells per well were plated to the upper chamber of the Transwell chamber with 10% FBS containing the medium at the bottom layer. Incubated for 12 h at 37°C, the upper layer of the membrane was scraped and stained with 0.5% Crystal Violet Staining Solution and photographed under a microscope. (b) The number of migrated cells was quantified per well under a microscope by averaging five random fields (*n* = 5, ^∗^*P* < 0.05). (c) Wound healing assays. After transfection and scratch, the cells were photographed and counted under a phase contrast microscope 12 h later. Yellow and red lines showed start and end (12 h) positions of rMSCs after scraping. (d) Quantitative results of wound healing assays. The scratch area was observed under a phase contrast microscope and photographed (*n* = 3, ^∗^*P* < 0.05). All data are presented as mean ± SD.

To investigate the TINCR-induced migration of rMSCs, lvTINCR and siTINCR were transfected on rMSCs, respectively. The results of Transwell invasion assay and wound healing assay showed that lvTINCR increased the migration of rMSCs obviously and the siTINCR group decreased, compared with the control group, respectively (Figures 1(a)–1(d)). The number of migrated cells on Transwell invasion assay was quantified per well under a microscope by averaging five random fields (*n* = 5, ^∗^*P* < 0.05). The scratch area was observed under a phase contrast microscope and photographed (*n* = 3, ^∗^*P* < 0.05). All data are presented as mean ± SD.

### 3.2. TINCR Sponged mir-761

**Figure 2:**
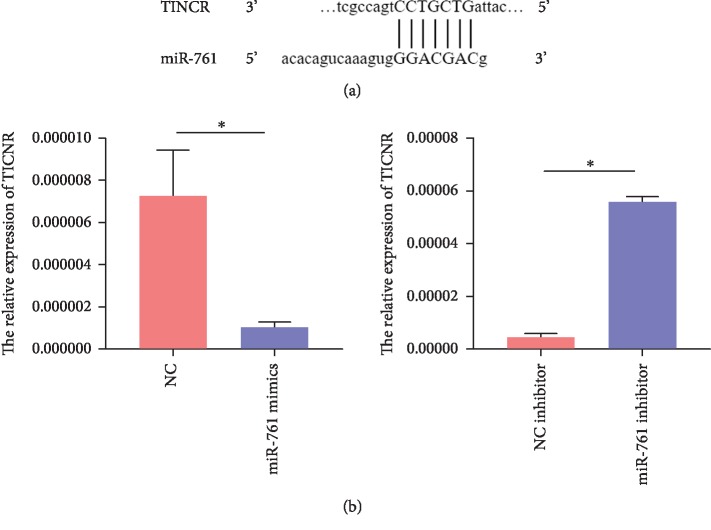
TINCR sponged miR-761. (a) Using the miRDB, we found that TINCR would be likely to sponge miR-761. (b) Further validation by RT-PCR revealed that miR-761 involves the expression of TINCR. Data are presented as mean ± SD (*n* = 3, ^∗^*P* < 0.05).

lncRNAs could function as ceRNA to sponge miRNAs resulting in preventing microRNAs from binding to their target genes. To explore the underlying mechanism, we found that TINCR would be likely to sponge miR-761 as predicted by miRDB. Further validation by RT-PCR revealed that miR-761 involves the expression of TINCR. miR-761 mimics upregulated the expression of TINCR, while miR-761 inhibitor downregulated the expression (Figures 2(a) and 2(b)). Data are presented as mean ± SD (*n* = 3, ^∗^*P* < 0.05). However, the function of TINCR and miR-761 on the migration of rMSCs is largely unknown, so further in vitro analysis of TINCR and miR-761 was conducted.

### 3.3. miR-761 Regulated Migration by Targeting Wnt2

**Figure 3:**
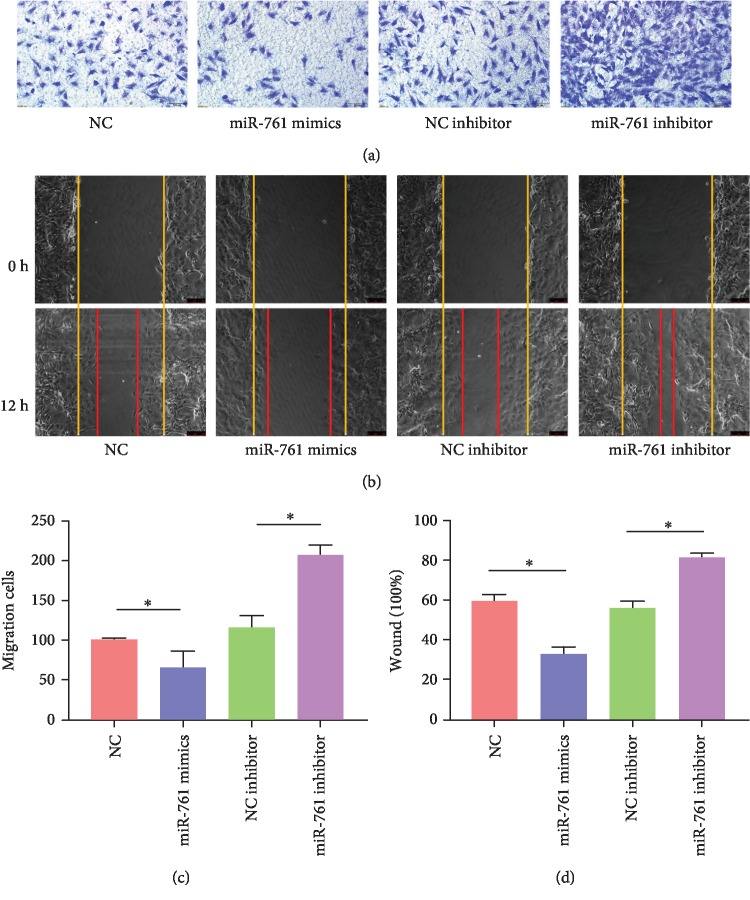
The migration ability of rMSCs was evaluated by Transwell assays and wound healing assays after transfecting NC inhibitor, miR-761 inhibitor, NC mimics, and miR-761 mimics. The NC inhibitor, miR-761 inhibitor, NC mimics, and miR-761 mimics were transfected on rMSCs, respectively. (a) Transwell assays. Cells with a density of 8 × 10^4^ cells per well were plated to the upper chamber of the Transwell chamber with 10% FBS containing the medium at the bottom layer. Incubated for 12 h at 37°C, the upper layer of the membrane was scraped and stained with 0.5% Crystal Violet Staining Solution and photographed under a microscope. (b) The number of migrated cells was quantified per well under a microscope by averaging five random fields (*n* = 5, ^∗^*P* < 0.05). (c) Wound healing assays. After transfection and scratch, the cells were photographed and counted under a phase contrast microscope 12 h later. Yellow and red lines showed start and end (12 h) positions of rMSCs after scraping. (d) Quantitative results of wound healing assays. The scratch area was observed under a phase contrast microscope and photographed (*n* = 3, ^∗^*P* < 0.05). All data are presented as mean ± SD.

To evaluate the effect of miR-761 on the migration of rMSCs, NC mimics, miR-761 mimics, NC inhibitor, and miR-761 inhibitor were also transfected into rMSCs, respectively. Based on the result (Figures 3(a)–3(d)), the miR-761 mimic group suppressed the migration ability of rMSCs distinctly, compared with the NC mimic group, whereas the miR-761 inhibitor group promoted the migration ability, compared with the NC inhibitor group. The number of migrated cells on the Transwell invasion assay was quantified per well under a microscope by averaging five random fields (*n* = 5, ^∗^*P* < 0.05). The scratch area was observed under a phase contrast microscope and photographed (*n* = 3, ^∗^*P* < 0.05). All data are presented as mean ± SD. And these data revealed that miRNA-761 negatively regulates migration of rMSCs.

**Figure 4:**
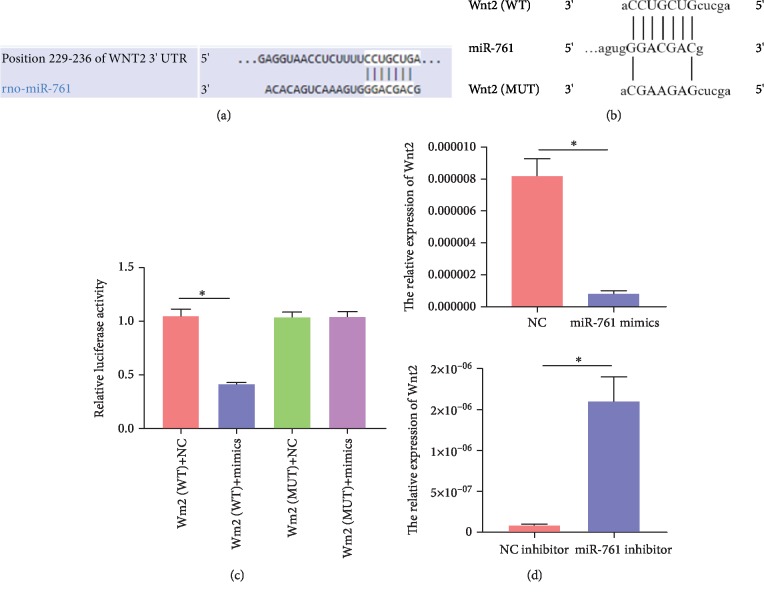
Wnt2 is a potential target of miRNA-761. (a) Using TargetScan for matching with potential target genes, we discovered that the migration-related Wnt2 is one of the targets with miR-761 binding site in its 3′UTR region. (b) We synthesized a wild-type 3′UTR (WT-Wnt2) with miR-761 binding site and a mutant 3′UTR (MU-Wnt2) without. (c) The luciferase reporter activity was remarkably inhibited in the group of WT-Wnt2 with miR-761 mimics, but not in the group of MU-Wnt2 with miR-761 mimics (*n* = 3, ^∗^*P* < 0.05). (d) Real-time PCR results revealed that miR-761 was a negative factor for Wnt2 expression in rMSCs (*n* = 3, ^∗^*P* < 0.05). All data are presented as mean ± SD.

To further explore the intracellular molecular mechanism by which TINCR regulates migration of rMSCs by sponging miR-761, we used TargetScan for matching with potential target genes. Fortunately, we discovered that the migration-related Wnt2 is one of the targets with miR-761 binding site in its 3′UTR region (Figure 4(a)). To identify whether miR-761 directly targets Wnt2, the luciferase reporter assay was conducted. We synthesized a wild-type 3′UTR (WT-Wnt2) with miR-761 binding site and a mutant 3′UTR (MU-Wnt2) (Figure 4(b)), then inserted them into the plasmid, respectively. The plasmid, as well as NC mimics or miR-761 mimics, was cotransfected into the 293T cell. The luciferase reporter activity was remarkably inhibited in the group of WT-Wnt2 with miR-761 mimics, but not in the group of MU-Wnt2 with miR-761 mimics (Figure 4(c)). Real-time PCR results revealed that miR-761 was a negative factor for Wnt2 expression in rMSCs (Figure 4(d)). All data are presented as mean ± SD (*n* = 3, ^∗^*P* < 0.05).

### 3.4. TINCR Regulated miR-761-Induced Migration of rMSCs

**Figure 5:**
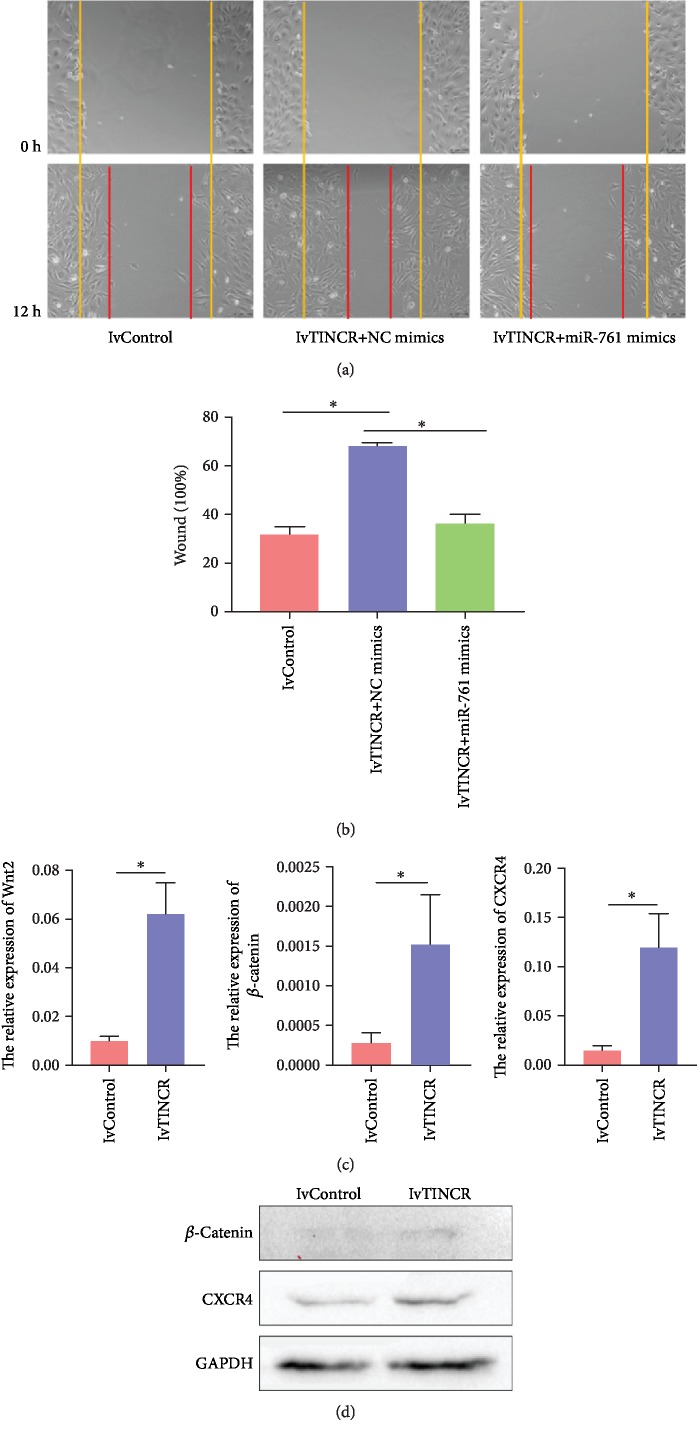
TINCR regulated miR-761-induced migration of rMSCs. (a) Wound healing assays. After cotransfection with miR-761 mimics and lvTINCR and scratch, the cells were photographed and counted under a phase contrast microscope 12 h later. Yellow and red lines showed start and end (12 h) positions of rMSCs after scraping. (b) Quantitative results of wound healing assays. The scratch area was observed under a phase contrast microscope and photographed (*n* = 3, ^∗^*P* < 0.05). All data are presented as mean ± SD. (c) Real-time PCR results revealed that the overexpression of TINCR could upregulate the level of Wnt2, *β*-catenin, and CXCR4 mRNA in rMSCs (*n* = 3, ^∗^*P* < 0.05). All data are presented as mean ± SD. (d) Western blot results revealed that the expression of *β*-catenin and CXCR4 was upregulated after transfecting with lvTINCR.

For the sake of further investigation of the function of TINCR in miR-761-induced migration of rMSCs, we then cotransfected rMSCs with miR-761 mimics or NC mimics with lvTINCR. As shown in Figures 5(a) and 5(b), miR-761 mimics reduced the promoting impacts of lvTINCR on the wound healing of rMSCs in vitro. The scratch area was observed under a phase contrast microscope and photographed (*n* = 3, ^∗^*P* < 0.05). These results suggested that TINCR regulated the miR-761-induced migration of rMSCs.

Next, we examined the levels of Wnt2, *β*-catenin, and CXCR4 expression in rMSCs transfected with lvTINCR. The results showed that overexpression of TINCR increased the expression of Wnt2, *β*-catenin, and CXCR4 (Figure 5(c)). The data are presented as mean ± SD (*n* = 3, ^∗^*P* < 0.05). And we also found that the levels of *β*-catenin and CXCR4 protein increased in rMSCs transfected with lvTINCR (Figure 5(d)).

In summary, our results demonstrated that TINCR took part in the regulation of miR-761-induced migration in rMSCs through the regulation of Wnt2 and its Wnt2 signaling pathway. These results proved that TINCR modulates migration of rMSCs through the mir-761/Wnt2 axis.

## 4. Discussion

Being one of the important classes of noncoding RNAs, the role of numerous lncRNAs has been identified in MSCs. For instance, lncRNA TCONS_00041960 [24] can target miR-204-5p and miR-125a-3p to enhance osteogenesis and inhibit adipogenesis. lncRNA-p21 [25], under hypoxic preconditioning, can be a positive factors for MSC migration and survival capacity. However, the role of lncRNA-TINCR in the migration of rMSCs is still unknown. This study investigated the mechanism involved in lncRNA-TINCR regulation of rMSC migration. Therefore, it is important to identify the positive or negative regulators of migration of rMSCs. Moreover, we also identified that miR-761 plays as a negative regulator in it. Further research showed that Wnt2 was a direct target of miR-761. These findings suggest that the TINCR/miR-761/Wnt2 signaling pathway is an important part of the regulatory machinery involved in the migration of rMSCs.

Interestingly, previous research showed that TINCR exerted discrepant function in different subjects. Some researchers found that a higher TINCR level is correlated with poor survival in non-small-cell lung cancer (NSCLC), gastric cancer (GC), colorectal cancer (CRC), hepatocellular carcinoma (HCC), and breast cancer, because TINCR promotes cell apoptosis and migration [5, 16, 26, 27]. Others reported that the overexpression of TINCR suppressed cell proliferation and invasion in lung cancer cells, tongue squamous cell carcinoma, and cutaneous squamous cell carcinoma. And about the role of TINCR in the migration of rMSCs [12–14], in this study, we first used real-time quantitative RT-PCR to validate whether miR-761 regulates the expression of TINCR. Then, we evaluated the effect of TINCR and miR-761 on the migration of rat MSCs from the bone marrow by Transwell assays and wound healing assays. Our data showed that TINCR is a positive regulator of migration of rMSCs. miR-761 mimics suppressed rMSC migration, while miR-761 inhibitor promoted it. These findings suggested that TINCR functions as a ceRNA by sponging miR-761, which has an impact on the migration of rMSCs.

To explore the intracellular molecular mechanism by which TINCR regulates migration of rMSCs by sponging miR-761, we used target gene prediction software, like TargetScan and miRDB, for matching with potential target genes which have an established function in promoting migration of rMSCs. Fortunately, we discovered that Wnt2 is one of the targets of miR-761. In this study, we found that transfection of miR-761 mimics decreases in cell migration obviously, whereas miR-761 inhibitor increased. The results are consistent with previous studies [28, 29]. Then, conducting dual-luciferase reporter gene assay, we revealed that overexpression of miR-761 mimics suppressed the luciferase activity of the reporter construct. Inversely, this effect was abolished by cotransfecting a mutant 3′UTR of Wnt2 and miR-761 mimics in rMSCs. Subsequently, in order to further investigate the function of TINCR in miR-761-induced migration of rMSCs, the result of wound healing assay on rMSCs cotransfected with miR-761 and lvTINCR shows that miR-761 mimics reduced the promoting impacts of lvTINCR. Meanwhile, the expression of Wnt2 and *β*-catenin was upregulated, which illustrated that TINCR could activate the Wnt signaling pathway via miR-761/Wnt2 axis to promote rMSC migration. In addition, the expression of cell migration-related proteins was assessed, such as C-X-C motif chemokine receptor 4 (CXCR4). Our result verified that TINCR could upregulate the level of CXCR4 mRNA and protein. CXCR4 encoded on chromosome 2 is a G protein-coupled chemokine receptor, which is well established that plays a crucial role in MSC homing [30–32]. Moreover, some researchers have also given a clue to describe the correlation between CXCR4 and Wnt signaling pathway on cell migration [33–35]. To sum up, this study proved that TINCR modulates migration of rMSCs through the mir-761/Wnt2 axis.

Given these findings, there are several important significances. Firstly, MSC homing is considered a natural self-healing response. This response can be stimulated by specific signals from injured tissue, resulting in MSCs leaving their niche and migrating to those injured tissues to proliferate and differentiate [21, 36]. Undoubtedly, further understanding of the mechanism of MSC migration is beneficial to stem cell therapy and regenerative medicine outcomes [37]. Secondly, the Wnt signaling pathway can regulate cell proliferation, differentiation, apoptosis, and migration [38]. Exactly, Wnt2, as an important member of the Wnt protein family, has been reported to be involved in tumorigenesis and progression through initiating the Wnt signaling pathway [39–41]. So, this study has raised the possibility that TINCR could modulate the role of the Wnt signaling pathway activated by Wnt2, through sponging miR-761, thereby controlling cancer progression. Moreover, Wnt2 is not the one and only target gene of miR-761. Extensive genes have been identified as the target of miR-761 in numerous cancers, including HDAC1, Rab3D, Runx3, Mitofusin-2, CXCR1, TRIM29, MSI1, ING4, TIMP2, and GSK3*β*. In brief, TINCR, as a ceRNA, has a potential capability of regulating these target genes of miR-761, which results in tumorigenesis positively or negatively [28, 29, 42–48].

In conclusion, our results demonstrate that lncRNA-TINCR functions as a competitive endogenous RNA (ceRNA) to promote the migration of rMSCs by sponging miR-761, which upregulates the expression of Wnt2. Inversely, this results in suppression of the Wnt2 signaling pathway. Thus, lncRNA-TINCR may serve as a potential target for enhancing MSC homing through the miR-761/Wnt2 signaling pathway. Even so, promoting rMSC migration by the interaction of CXCR4 and the Wnt signaling pathway which is initiated by the Wnt2 protein, the underlying mechanism of it still remains elusive.

## Figures and Tables

**Table 1:** Primers for qRT-PCR.

Primer name	Sequence (5′-3′)
TINCR forward TINCR reverse	TCTTGGCCTTTGGAACCAGG ACGCTAAGGTTGTCCGTCTG
Wnt2 forward Wnt2 reverse	CATCGCTGGAACTGCAACAC ATCTACAAATGCACGGGCGA
*β*-Catenin forward	CCCCTGCAACGATCTGACT
*β*-Catenin reverse	TTGCTCTTGCGTGAAGGACT
CXCR4 forward	AGTGACCCTCTGAGGCGTTT
CXCR4 reverse	TTGCCCACTATGCCAGTCAA

## Data Availability

The data used to support the findings of this study are available from the corresponding author upon request.
